# High-density microelectrode array recordings and real-time spike sorting for closed-loop experiments: an emerging technology to study neural plasticity

**DOI:** 10.3389/fncir.2012.00105

**Published:** 2012-12-20

**Authors:** Felix Franke, David Jäckel, Jelena Dragas, Jan Müller, Milos Radivojevic, Douglas Bakkum, Andreas Hierlemann

**Affiliations:** Department of Biosystems Science and Engineering, ETH ZürichBasle, Switzerland

**Keywords:** closed-loop, real-time, spike sorting, multielectrode arrays, neural cultures

## Abstract

Understanding plasticity of neural networks is a key to comprehending their development and function. A powerful technique to study neural plasticity includes recording and control of pre- and post-synaptic neural activity, e.g., by using simultaneous intracellular recording and stimulation of several neurons. Intracellular recording is, however, a demanding technique and has its limitations in that only a small number of neurons can be stimulated and recorded from at the same time. Extracellular techniques offer the possibility to simultaneously record from larger numbers of neurons with relative ease, at the expenses of increased efforts to sort out single neuronal activities from the recorded mixture, which is a time consuming and error prone step, referred to as spike sorting. In this mini-review, we describe recent technological developments in two separate fields, namely CMOS-based high-density microelectrode arrays, which also allow for extracellular stimulation of neurons, and real-time spike sorting. We argue that these techniques, when combined, will provide a powerful tool to study plasticity in neural networks consisting of several thousand neurons *in vitro*.

## Introduction

The understanding of neural circuits and their activities is to a major extent based on measurements with extracellular electrodes. This is due to the fact that extracellular recordings are relatively easy to perform and very well established. In contrast to single cell measurements with intracellular recording techniques, extracellular electrodes pick up the action potentials (spikes) of all neurons in their vicinity. This is a blessing as well as a curse. An advantage is that in principle several neurons can be measured simultaneously using a single extracellular electrode, but the price to pay is the need to assign single spikes to their putative neuronal sources. This problem is referred to as spike sorting and it is known to be difficult and error-prone (Lewicki, 1998), and spike sorting often involves a highly time consuming, manual component.

**Figure 1:**
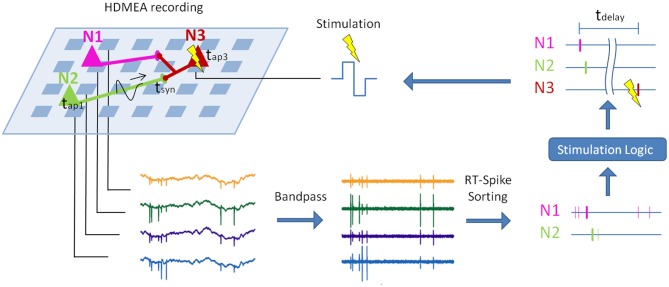
**Principle of real-time closed-loop experiments with spike sorting.** Sketch of a potential real-time closed-loop stimulation on an HDMEA, combined with spike sorting. The electrical activity of three neurons (colored triangles) is measured by a high-density array of electrodes (light blue squares). First, the recorded signal is bandpass-filtered. In a second step, spike sorting is applied to compute the spike times of the single neurons. Depending on the sorted spike trains and the stimulation logic, the postsynaptic neuron (N3) is stimulated (Müller et al., in review). If the stimulation latency (*t*_delay_) is short enough, the stimulation can be timed with respect to the arrival of the action potentials of N1 and N2 at their synapses to N3 (*t*_syn_). This can be used to change the synapse characteristics via spike-timing-dependent plasticity (Feldman, 2012). Parts of this graph were adopted from Einevoll et al. (2011).

Depending on the experiment, time consuming spike sorting can be regarded as a mere inconvenience, and many studies have focused on the development of spike sorting algorithms for the offline analysis of the recordings after performing the experiment (see e.g., Letelier and Weber, 2000; Shoham and Fellows, 2003; Delescluse and Pouzat, 2006). For real-time closed-loop experiments and brain machine interfaces (BMI), however, it is absolutely necessary to obtain spike trains already during the recording so that time consuming spike sorting is not only a problem but essentially prohibits performing such experiments. Therefore, spike sorting is usually avoided in those experiments by detecting just the presence of action potentials, e.g., by applying a voltage threshold, which can be relatively easy and efficiently implemented also in hardware (Guillory and Normann, 1999). Real-time spike detection allows for studying closed-loop feedback of neural activity, for example, through the implementation of visual feedback to an awake monkey (Fetz, 1969), or by applying electrical stimulation to neurons in an awake animal (Jackson et al., 2006). Electrical stimulation of neurons that depends on the activity of other neurons (see also Figure 1) was also successfully used in neural cultures on top of multi-electrode arrays (MEAs): electrical feedback stimuli have been used to control the bursting activity of cultured neurons in Wagenaar et al. (2005) and the connection strengths between neurons in Müller et al. (in review). The closed-loop approach can also be used to connect a neural network to a robot (Bontorin et al., 2007; Potter, 2010). For a review of real-time closed-loop electrophysiology see, e.g., Arsiero et al. (2007). These studies, however, were all realized without using spike sorting, either by limiting the number of single neurons that were recorded from (by trying to detect only one specific neuron per electrode), or by using multi-unit activities.

Recent developments in measurement techniques and in spike sorting algorithms make it now possible to overcome some of the limitations of extracellular recordings. A possible setup using spike sorting for closed-loop stimulation of specific neurons is shown in Figure 1. To use the closed loop, e.g., to investigate spike-timing-dependent plasticity, the real-time spike-sorting-induced latency may not exceed a few milliseconds. In the following, we will review the advances in MEA recording technology with a special focus on high-density MEAs and show that the high-density of the electrodes provides unprecedented signal quality that holds the promise to enable clear and reliable assignment of single spikes to putative neurons (Litke et al., 2004; Prentice et al., 2011; Jäckel et al., 2012).

## MEA recording technology

Planar MEAs are two-dimensional arrangements of recording electrodes for *in vitro* extracellular measurements of cultured neuronal cells or slice preparations. They allow for recording of electrical activity simultaneously on many electrodes at high temporal resolution. Thus, they represent an important tool to study the dynamics in neuronal networks (e.g., Potter et al., 2006; Bontorin et al., 2007; Chao et al., 2007; Rolston et al., 2010; Müller et al., in review).

An important parameter of MEAs is the inter-electrode distance (IED). For multi-electrode arrangements on shafts of needles, such as tetrode configurations (Eckhorn and Thomas, 1993; O'Keefe and Recce, 1993), this distance is small enough (less than 20 μm) that a single action potential can be simultaneously detected on several electrodes. The maximal distance between a neuron and an electrode, at which the action potentials of the neuron can be still measured, is assumed to be smaller than 50–70 μm although this greatly depends on the recording setup and the respective preparation (Buzsáki, 2004; Frey et al., 2009b). For traditional, commercially available MEAs, however, the IED was usually much larger [100–200 μm IED and 60–200 metal electrodes on a glass substrate (Stett et al., 2003)] so that MEA recordings constituted, in principle, multiple simultaneous single-electrode recordings. In other words, the distance between the electrodes was too large to detect activity of the same single neuron on multiple electrodes.

From the signal processing point of view, this is an unfavorable recording situation, as recording the same action potential with more than one electrode was shown to strongly increase spike sorting performance (Gray et al., 1995). Furthermore, many neurons will lie in between electrodes and not be measured at all. To ensure that neurons lie close to the electrodes, additional measures can be taken during the preparation of the cultures, such as patterning the cells at electrode locations (Shein et al., 2009), but this adds complexity to the experimental procedure.

Recent advances in microtechnology, especially the realization of MEAs in complementary metal–oxide–semiconductor (CMOS) technology (Berdondini et al., 2009; Lambacher et al., 2010; Hierlemann et al., 2011), made it possible to greatly increase the number of electrodes per MEA, for example to 4096 in Berdondini et al. (2009), 11,011 in Frey et al. (2010) or 16,384 in Lambacher et al. (2010), while decreasing the IED to less than 20 μm, a distance comparable to that of the previously mentioned electrode ensembles on needles (e.g., tetrodes). Additionally, this technology provides increased signal quality through on-chip amplification and digitization circuits. Using on-chip multiplexing schemes, high-density MEAs (HDMEA) systems have been realized, which enable to read out large numbers of electrodes, arranged at high spatial density (Eversmann et al., 2003; Berdondini et al., 2005; Hutzler et al., 2006; Frey et al., 2009a).

**Figure 2:**
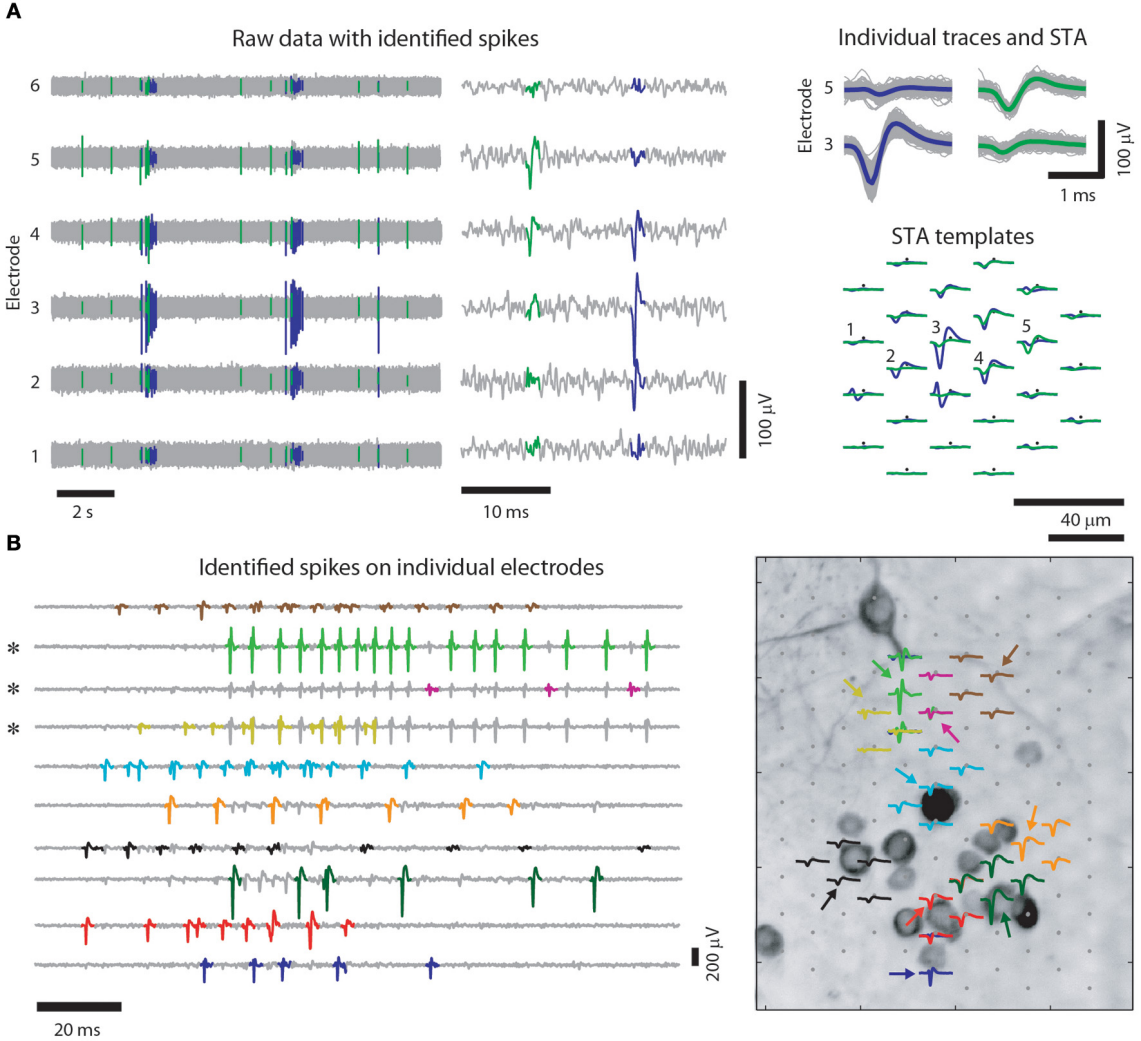
**Spike sorting for high-density multi-electrode recordings of cultured neurons. (A)** Example recording of 6 out of 102 electrodes of a HDMEA (left), where mainly two neurons were recorded from, and a close up on two spikes (middle) (similar figure as in Frey et al. (2009a), however, with cultured cortical neurons). Spikes of individual neurons are recorded by multiple electrodes. Colored traces are identified spikes from two neurons. Note that on the trace of electrode 4, the two spikes are hardly distinguishable and that only combining the information of different channels enables unambiguous spike assignment, see also (Fiscella et al., 2012). (Right top) Several superimposed spike traces of the two neurons. The colored traces are the spike-triggered averages (STAs) of the two neurons on the respective electrodes. The templates of the two neurons (green and violet) spatially overlap (right bottom) indicating that the same set of electrodes recorded from both neurons. **(B)** Spikes (left) and templates (right) for 10 identified neurons (colored traces). For each neuron, the electrode was chosen, where its template had the largest peak-to-peak amplitude (indicated by the colored arrows in the right panel). Note that some of the spikes are visible on more than one electrode (three channels marked by asterisks) and that high-amplitude spikes on one electrode can overlap with spikes on another electrode. Right: for illustration purposes the identified templates are superimposed onto a MAP2 staining of the culture they were recorded from Bakkum et al. (in review). Note that the electrodes have a similar IED than the distance between neurons.

The closely spaced microelectrodes of HDMEAs enable that virtually every neuron on the array is detected by multiple electrodes. Along with the additional information where the signal originated from, the high electrode density greatly improves spike sorting (Gray et al., 1995; Harris et al., 2000; Einevoll et al., 2011; Prentice et al., 2011). Figure 2 shows an example of such a recording.

However, HDMEAs do not only improve recording but also stimulation capabilities. Localized, reliable stimulation of single cells (Hottowy et al., 2012) is a powerful tool for plasticity experiments (Müller et al., in review). Indeed, sub-cellular sized electrodes have been shown to provide reliable stimulation of individual neurons *in vitro*. This has been demonstrated using MEAs with particularly high electrode densities that feature only stimulation capabilities, such as (Braeken et al., 2010; Lei et al., 2011). Procedures how to optimally stimulate a given neuron by using multiple electrodes and complex stimulation patterns are currently under investigation.

HDMEAs featuring recording and stimulation circuitry (Frey et al., 2010; Eversmann et al., 2011) combine the advantages of reliable spike sorting and localized single neuron stimulation, which paves the way to truly bidirectional experiments on single-cell level within the network context.

## Real-time spike sorting algorithms

The overall spike sorting process consists of a number of non-trivial processing steps (for a schematic of the spike sorting process see, e.g., Einevoll et al., 2011). First, spikes need to be detected in the noisy signals. For multi-electrode-shaft and HDMEA recordings, a single action potential can be detected on multiple electrodes. Then, a short piece of data is usually cut out around the detected events (potentially on multiple electrodes) and structured into a vector in a high dimensional space. Spike features are then extracted from this piece using, e.g., principle component analysis (Lewicki, 1998). This step aims at reducing the dimension of the vector space in order to keep dimensions that carry most information about the origin of the spikes and to remove dimensions that only carry noise. The goal of the feature space representation and dimensionality reduction is that spikes from the same neuron, i.e., appear to be similar to each other, are located closely together while being distant from spikes of other neurons. The most demanding step, achieved by using a clustering routine, is to determine how many neurons were recorded from, and which spike was produced by which neuron. Since most standard spike sorting procedures (e.g., Harris et al., 2000; Shoham and Fellows, 2003; Quiroga et al., 2004) need to store all individual spikes before the clustering step, they are not applicable for online spike sorting with the notable exceptions of Öhberg et al. (1996), where a neural network is used for real-time spike sorting, and (Rutishauser and Schuman, 2006), where the clusters are formed in an online procedure. The output of the spike sorting consists of the number of neurons, the individual neuronal spike trains, and the prototypic spike waveforms (called templates) for every neuron.

Since some data from a certain preparation can already be recorded and stored prior to a specific experiment, templates can be pre-computed using an offline spike sorter. This way, fast and efficient classifiers can be designed based on stored templates that are able to sort spikes in real-time. It does not come as a surprise that almost all research efforts in the direction of real-time spike sorting follow this approach (Friedman, 1968; Mishelevich, 1970; Roberts and Hartline, 1975; Stein et al., 1979; Salganicoff et al., 1988; Yang and Shamma, 1988; Gozani and Miller, 1994; Santhanam et al., 2004; Asai et al., 2005; Takahashi and Sakurai, 2005; Vollgraf et al., 2005; Biffi et al., 2010; Franke, 2011), although not all of these approaches explicitly make use of templates to derive spike classifiers.

So far, real-time spike sorting was mainly achieved by deriving simple hardware-implementable decision rules, based on the spike templates. One such rule is to check, if the spike voltage sample at a given time lies between a lower and an upper threshold relative to the peak of the spike waveform (a so called hoop), as described in Santhanam et al. (2004). Such decision rules are also used in commercially available recording systems and were individually applied to single electrodes (Nicolelis et al., 1997; Wessberg et al., 2000; Taylor et al., 2002; Guenther et al., 2009).

However, there have been only few applications of these approaches to multielectrode arrays in real-time scenarios, such as Takahashi and Sakurai (2005), where independent-component analysis was used to separate individual neuronal activities. The information of several recording channels must be efficiently combined for multi-electrode recordings. Extending a spike sorting method that works for single electrodes to multi-electrodes is not a trivial task and might not be possible for all methods.

As already discussed, HDMEAs impose even higher demands on the methods due to the large overall number of simultaneously recorded neurons and the large number of electrodes that are available per single neuron. There are a number of approaches to spike sorting of HDMEA data (Meister et al., 1994; Litke et al., 2004; Jäckel et al., 2011, 2012; Prentice et al., 2011; Fiscella et al., 2012) but none of those has been evaluated with respect to low latency real-time spike sorting so far. There is also no commercial system with real-time spike sorting available, and it is currently unclear how effective the application of the “hoop”-approach (Santhanam et al., 2004) is. Another ICA-based real-time approach has been described in Takahashi and Sakurai (2005), but the performance of ICA to separate all neurons of HDMEA data sets was found to be limited (Jäckel et al., 2012).

### Linear filters for spike sorting

Linear-filter-based spike sorting approaches rely on linear filters that preferentially respond to one template that is considered to represent spikes from a single neuron (Roberts and Hartline, 1975; Stein et al., 1979; Gozani and Miller, 1994; Vollgraf and Obermayer, 2006; Franke et al., 2010; Franke, 2011). Spikes can then be detected by thresholding the filter outputs. An alternative method was suggested in Vollgraf et al. (2005), where a preprocessing filter was designed to be tuned to the average spike waveform of all spikes. However, detected spikes have subsequently to be clustered in the filter output space, which introduces a complex problem after the filtering. Filter-based methods hold the promise to be suitable even for low-latency real-time spike sorting of MEA: linear filters can be efficiently implemented in hardware and they scale well with the number of recording electrodes. Firstly, all electrodes can be processed in parallel, and, secondly, if spikes of one neuron cannot be detected on a given electrode, this electrode can be ignored for the corresponding filter (Jäckel et al., 2011).

It was argued that linear-filter-based spike sorting provides only moderate performance in terms of sorting quality (Wheeler and Heetderks, 1982; Lewicki, 1994; Guido et al., 2006), but it was shown more recently that this could be due to the fact that the candidate filters have been derived in the frequency domain, which was shown to be non-optimal (Vollgraf and Obermayer, 2006).

### Real-time implementation

Numeric computations behind linear filters are based on multiply-accumulate (MAC) operations. For every recording electrode, a set of filter coefficients has to be multiplied with the most recent samples of the recordings, and all multiplications over all electrodes are then summed up. Since multiplications are independent of each other, they can be done in parallel on a digital signal processor (DSP) as a single processing step. DSPs are well suited for implementing MAC-based algorithms, but filter-based spike sorting algorithms can consist of more complex operations [like buffering the filter outputs, thresholding, and estimation of the filter with the maximal output (Franke, 2011)], which requires more flexibility than provided by DSPs. Such more complex operations can, however, be implemented by using field-programmable gate arrays (FPGAs). The digital interface of a MEA can be controlled by these fast and reprogrammable microcontrollers. By integrating data analysis modules, as well as stimulation logics directly on the FPGA, the complete closed-loop experiment can be realized in “programmable hardware” (Hafizovic et al., 2007). This obviates the necessity to route the signal path through a PC, which would increase latency and jitter. Another advantage of FPGAs is the relatively large available memory to store filter coefficients.

### Overlapping spikes

When two spikes occur nearly at the same time, they can cause problems for the spike sorting: The overlapping signals could be detected as a single spike instead of being recognized as two spikes, and the distorted overall waveform can lead to misclassifications. With multi-electrode recordings, there can be two different types of spike overlaps: (1) temporal overlaps include spikes that occur nearly at the same time but on different electrodes, while (2) spatio-temporal overlaps occur nearly at the same time and also on the same electrodes. Purely temporal overlaps do not cause any problems for filter-based methods, as the filters corresponding to one neuron can be made “blind” to the electrodes of another neuron and can be treated separately. Spatio-temporal overlaps (see Figure 2), however, will distort the filter outputs of both filters. A way to solve this problem is to remove the corresponding waveform from the data, once a spike was detected, and to then re-compute the filter outputs (Gozani and Miller, 1994; Franke, 2011). This approach is not well suited for a challenging real-time implementation, since it will generate a larger delay for overlapping spikes than for non-overlapping ones. The realization of an efficient overlap resolution technique for high-electrode-density data of real-time applications is still an open issue.

## Discussion/outlook

A number of issues in implementing real-time spike sorting still remain unsolved. It would be desirable to make the linear filters as short as possible to achieve the smallest possible delay (the delay of a causal filter is directly related to its length) (Vollgraf and Obermayer, 2006). However, it was not investigated yet, how short the filters for HDMEA recordings can be, while still ensuring a high spike sorting quality. Furthermore, the filters described in Roberts and Hartline (1975) are, in principle, more powerful than a simple matched filter (Vollgraf et al., 2005; Franke, 2011), since they try to suppress spikes from other neurons. This may be useful to resolve overlapping spikes but comes at a price: the filters might be less robust to noise, since they are under stronger constraints. Additionally, spike waveforms of two different neurons may not necessarily be linearly independent, which poses a problem for this kind of linear filters.

Given the high spatial resolution of HDMEAs, it will be interesting to investigate, how the quality of the results obtained by using simple spike sorting algorithms compares to that of more complex ones. Promising algorithms for use with high electrode density include the aforementioned “hoop”-approach (Santhanam et al., 2004), or a sorting that is solely based on the identities of the electrodes, on which a spike was detected.

An important issue for spike sorting is the occurrence of bursts. Here, a neuron produces potentially many spikes with successively decreasing amplitudes and, possibly, varying waveforms (Fee et al., 1996). For most algorithms, it is not known, how the spike sorting error rate is affected by bursts. HDMEAs seem to offer the potential to correctly sort spikes according to their relative amplitude distribution over many electrodes, which may be a robust feature also preserved during bursts (Rinberg et al., 1999).

HDMEAs are a valuable tool to study neural networks, and in combination with real-time spike sorting, hold great promise for new closed-loop experiments to study, e.g., neural plasticity. We have discussed the potential applicability of spike-sorting algorithms for this purpose and come to the conclusion that the combination of hardware-optimized algorithms with HDMEA recordings may possibly enable high performance spike sorting of more than hundred neurons with latencies in the range that is required to stimulate and control synaptic plasticity (Feldman, 2012). This may allow for experiments similar to those reported in Fetz (1969); Jackson et al. (2006); Bontorin et al. (2007); Rebesco et al. (2010), however, with the possibility to use sophisticated feedback stimuli upon occurrence of defined signature signals of single neurons within a local population.

### Conflict of interest statement

The authors declare that the research was conducted in the absence of any commercial or financial relationships that could be construed as a potential conflict of interest.
